# Coronary Artery Bypass Grafting: A Precipitating Factor for Perioperative Diabetic Ketoacidosis

**DOI:** 10.5812/ijem.7183

**Published:** 2013-04-01

**Authors:** Vishal Sehgal, Sukhminder jit Singh Bajwa, Abbas Kitabchi

**Affiliations:** 1 Regional Hospital of Scranton, The Commonwealth Medical College Scranton, PA, USA; 2 Department of Anaesthesiology and Intensive Care, Gian Sagar Medical College and Hospital, Banur, Patiala, Punjab, India; 3 Department of Medicine and Endocrinology, A202 Coleman College of Medicine Building, Court Avenue, Memphis TN, USA

**Keywords:** Coronary Artery Bypass Grafting, Diabetes Mellitus, Diabetic Ketoacidosis, Insulin

## Abstract

Non-Insulin Dependent Diabetes Mellitus (NIDDM) is a common disease entity in patients with Coronary Artery Disease (CAD). Diabetic Ketoacidosis (DKA) is not only one of the major complications of Diabetes Mellitus but also a significant challenging clinical entity for the patients undergoing any elective or emergency surgery. Coronary Artery Bypass Grafting (CABG) being done in a patient with DKA has not been reported. We are presenting a rare case with DKA in whom CABG was carried out in a hospital devoted exclusively to cardiac cases. Insulin was given in very large doses as a part of therapeutic regimen and the outcome was favorable. This report concludes that if a patient undergoing urgent cardiac surgery incidentally develops DKA after induction of anesthesia, then the operation can be carried out provided DKA is managed aggressively. Also, major stress factors like cardio pulmonary bypass (CPB) and hypothermia should be avoided and care should be taken to avoid cerebral edema.

## 1. Introduction

Diabetic patients invariably pose numerous clinical challenges during any elective or emergency surgery. The clinical scenario becomes more challenging if such patients develop diabetes related complication during these therapeutic measures. We also encountered a rare clinical scenario where a patient with unstable angina (USA) and NIDDM on insulin infusion and maintaining high blood sugars developed DKA after induction of anesthesia. DKA was managed successfully and the patient was extubated next morning. There is no such case report in our knowledge when CABG was carried out in a patient with DKA.

## 2. Case Report 

A 56 year old woman, wt. 67 kgs and height 162cms, was admitted with complaints of uneasiness in the chest. Electrocardiogram (ECG) showed ST changes in II, III and aVF and ST in aVL, V_2_ - V_6_. The patient was a known case of NIDDM for 6 years and was being managed with oral hypoglycemic and had no previous history of CAD or Hypertension. HbA1c was 9.6 on admission. Following admission, she developed an episode of Ventricular Tachycardia (VT) followed by asystole. Trans-venous pacing (TPI) was done along with other resuscitative measures. Subsequently, angiography showed Triple Vessel Disease (TVD) and a moderately decreased LV Function.

Preoperatively the patient was on intravenous (i/v) regular insulin protocol in the ICU as was the norm in the institute. Accucheks were done two-hourly and urine was tested for ketones twice a day. On the day of the operation 6U of Lispro Insulin was given subcutaneously in the morning as FBS was 180 and patient was premedicated with Tab. Larpose, 2 hrs before the operation and Morphine and Promethazine were administered I/M 1 hour before surgery. After securing all the arterial and venous cannulae under local anaesthesia, the patient was pre-oxygenated for 5 minutes. Induction of anaesthesia was done with Thiopentone, Morphine, Isoflurane and Vecuronium Bromide. There was a lapse of 4 hours between stopping i/v insulin and induction of anesthesia.

Post-induction, accuchek showed a glucose of462 mg% and the ABG showed metabolic acidosis with S.HCO_3_ = 15 mmol/L, BE = -11.8mmol/L and pH = 7.249. Urine for ketone bodies was 3+. Insulin Lispro 10 U I/V were administered along with 25 mL of sodium bicarbonate solution (7.5%). Serum Potassium (K^+^) was found to be normal (4.2mmol/L). Repeat ABG after 30min showed pH = 7.23 and SHCO_3_ = 16mml/L and normal level of blood gases (Table 1). The patient was again given a bolus 10U insulin I/V as RBS was 398mg%. Following this intervention, basal insulin infusion was maintained at l0U/hr so as to keep RBS between 100-200mg%. It was decided to go ahead with the surgery and operate upon the patient off pump on a beating heart (OPCAB) to avoid further complications of CPB. The entire surgery was uneventful and she received 3 grafts.

The post-operative stay in the Intensive Care Unit (ICU) was also uneventful and the patient was extubated next morning. Her insulin requirement post operatively averaged 2U/hr and her RBS ranged between 118-170mg%. The patient was discharged from the hospital 5 days later.

**Table 1.** Showing Serial ABG, RBS, K+ and Insulin Requirement Intraoperatively

Time	pH	SHCO3, mmol/L	PO2, mmHg	PCO2, mmHg	K+, meq/L	RBS, mg/dL	Insulin Infusion, U/hr	InsulinI/V Bolus
**11.24am**	7.25	15.1	148	33	4.2	462	10	10U
**11.55pm**	7.53	17.1	155	39	3.9	398	10	10U
**12.32pm**	7.28	18.1	181	40	3.7	395	10	10U
**13.09pm**	7.35	21.3	178	38	3.7	304	10	10U
**13.38pm**	7.35	21.6	181	38	3.7	315	10	10U
**14.32pm**	7.43	24.1	148	35	3.6	245	10	---
**15.12pm** ** (post Op.)**	7.41	24.4	90	37	3.4	194		---

## 3. Discussion

Uncontrolled Diabetes Mellitus invariably poses numerous difficulties and complicated clinical scenarios during emergency surgeries. A typical picture of DKA attributable to anesthesia and surgery related stresses were encountered in the present case.

The three main primary modalities of treating DKA are fluid resuscitation, insulin infusion and correction of electrolyte and acid-base imbalance (1). In this case, only 700mL of Ringer's Solution was given and hemodynamics was maintained well with central venous pressure (CVP) ranging between l1-16mmHg. Insulin bolus doses were given repeatedly along with a baseline infusion until blood sugar came below 200mg%. CPB was avoided since it is established that it results in cerebral edema due to increased Blood Brain Barrier (BBB) permeability (2, 3). Mannitol 100mL (20%) was administered prophylactically to avoid any possible development of cerebral edema, the cause of which remains uncertain. Cerebral edema in DKA is responsible for causing a high mortality (3).

The total urine output during the peri-operative period was estimated at 100mL. Anesthesia, Surgery, hypothermia and CPB conventionally considered the major stress factors in the metabolic and hormonal responses to cardiac surgery (4, 5). To avoid the stress of hypothermia patient was kept off the pump. Hypothermia suppresses insulin secretion and is a more potent factor compared to anti-insulin hormones. Although, the patient's preoperative Insulin requirement was 40 U/ day, about 100U of insulin intra-operatively had to be given to control DKA over a period of 3 hrs.

Rapid response to the treatment of DKA could be achieved because the major stress factors including systemic hypothermia and the inflammatory response because of stress of CPB was avoided. The use of adequate anesthesia and sedatives including Fentanyl infusion were also contributory in controlling the subsequent stress response and a further rise in blood sugar.

In conclusion, we report a case of DKA during CABG, which was managed successfully. When DKA occurs due to anesthesia and surgery induced stress, high dose of insulin remains the treatment of choice. The stress of surgery during CABG can be minimized by avoiding CPB, hypothermia and also by reducing the duration of the surgical procedure. Also DKA could have been prevented if long acting insulin was used prior to stopping i/v insulin.
